# A short-term sublethal oral exposure to microcystin-LR disrupts cecal microbiome homeostasis in mallard

**DOI:** 10.3389/ftox.2025.1634241

**Published:** 2025-10-15

**Authors:** Serguei V. Drovetski, Valerie I. Shearn-Bochsler, Erik K. Hofmeister, Natalie K. Karouna-Renier, Robert J. Dusek

**Affiliations:** ^1^ U.S. Geological Survey, Eastern Ecological Science Center at the Patuxent Research Refuge, Laurel, MD, United States; ^2^ U. S. Geological Survey, National Wildlife Health Center, Madison, WI, United States

**Keywords:** microcystin-LR, mallard, cecum, microbiota function, Anna Karenina principle, metatranscriptomics

## Abstract

**Introduction:**

The frequency of cyanobacterial blooms seems to have increased globally in recent decades due to human induced eutrophication and climate change. Cyanobacterial blooms can produce several groups of toxins, among which microcystin-LR (MC-LR) is one of the most abundant. Effects of MC-LR on avian microbiome have not been studied and studies in laboratory murines have been limited to metabarcoding of prokaryotes.

**Methods:**

Using RNA shotgun sequencing, we compared the richness and composition of metabolically active prokaryotes, expressed virulence factors, antimicrobial resistance genes, metabolic pathways, Gene Ontology terms, enzymes, and proteins in mallards (Anas platyrhynchos) that were orally exposed to a sublethal dose of MC-LR for one week and unexposed birds.

**Results:**

Richness and composition of all compared features did not differ between exposed and control birds and none were differentially expressed between exposure groups. However, richness and/or composition of all features except virulence factors and Carbohydrate Active enzymes had multiple-fold greater dispersion in exposed birds than in controls. This effect was especially pronounced in expressed metabolic (MetaCyc) pathways.

**Discussion:**

Our results suggest that MC-LR exposure had a stochastic (rather than deterministic) effect on cecal microbiota, especially its function. Observed disturbance of the microbiota homeostasis is consistent with the Anna Karenina Principle. This principle has been documented in a wide range of eukaryotes using primarily microbial community metabarcoding. Although stochastic disturbance of microbiota function has been hypothesized, our study seems to be the first to demonstrate this in an experimental study.

## 1 Introduction

During recent decades, the frequency of harmful cyanobacterial blooms seems to have increased in fresh and brackish waterbodies globally (Paerl and Paul, 2012; Buratti et al., 2017; Anderson et al., 2021). Increasing anthropogenic eutrophication and global climate change synergistically facilitate expansion of cyanobacterial blooms (Paerl et al., 2011; Paerl and Paul, 2012; Michalak et al., 2013). Microcystin (MC) is the most frequently detected group of toxins produced by cyanobacteria (Campos and Vasconcelos, 2010; Li et al., 2017). They are potent hepatotoxins and among the >250 currently known congeners (Rattner, 2022), MC-LR is the most frequently reported (Gupta et al., 2003).

MC-LR toxicity has been well studied in laboratory mice (House mouse, *Mus musculus*). The intraperitoneal median lethal dose (LD50) for MC-LR in adult VAF/plus CD-1 and BULB/c mice was estimated at 0.065 mg/kg (Robinson et al., 1989; Yoshida et al., 1997), whereas the 24-h oral LD50 was estimated to be over two orders of magnitude higher - 10.9 mg/kg (Yoshida et al., 1997). Avian toxicity of MC-LR is less well known and LD50 concentrations have been estimated only for intraperitoneal injections but not for oral exposure. Intraperitoneal 24-h LD50 estimates for adult mallards (*Anas platyrhynchos*; 0.085 mg/kg body weight; mix of 96.4% MC-LR and 3.6% MC-YR) and week-old ducklings (0.065 mg/kg body weight) were similar to those of mice (Li et al., 2012). By more environmentally realistic oral administration, an acute outcome has not been achieved regardless of the dosage used. The highest concentration of MC-LR in these failed attempts to induce acute toxicity in mallards is reported at 17.5 mg/kg (Bong, 2021). Notwithstanding the failure to induce an acute outcome in oral exposure experiments, studies universally reported sub-lethal effects of MCs on different avian organs and systems, including liver, kidney, spleen, gut, and reproductive and immune systems (Pašková et al., 2008; Damkova et al., 2009; Damkova et al., 2 011; Peckova et al., 2009; Kral et al., 2012; Ondracek et al., 2012).

The great difference in outcomes between intraperitoneal injection of MCs and their oral administration suggests that the avian digestive system and microbiota may play an important role in MCs metabolism, especially in species that have well-developed ceca, such as galliforms and herbivorous waterfowl. The paired intestinal ceca play a crucial role in fowl adaptation to extracting nutrients from otherwise indigestible plant foods (Remington, 1989). They serve as microbial fermentation chambers in which non-starch polysaccharides (dietary fiber) are broken into volatile fatty acids (Clench and Mathias, 1995; Svihus et al., 2013) that can be utilized by the host as an energy source (Bergman, 1990). Furthermore, cecal microbiota are involved in detoxification of plant secondary compounds (Kohl et al., 2016) and environmental toxins (Jeong et al., 2013). In a study of the greater sage-grouse (*Centrocercus urophasianus*), the greatest seasonal differences in microbiota richness and composition related to dietary shifts from primarily insects and forbs in the summer to chemically defended sagebrush (*Artemisia* sp.) leaves in the winter were observed in the cecum (Drovetski et al., 2019). Likewise, during a chronic MC-LR exposure experiment with male BALB/c mice, the greatest changes in microbiota richness and abundance of sentinel bacteria were observed in the cecum (Chen et al., 2015). Given that gut microbiota plays a crucial role in its host’s health, immunity, and protection against pathogens (Hooper et al., 2012; Liang et al., 2018), a full understanding of MCs’ effects on the host is unlikely to be achieved without elucidating their effects on the gut microbiota. The authors are not aware of studies reporting effects of MC-LR exposure on avian microbiome.

Several experimental studies reported effects of MC-LR exposure on prokaryotic microbiota of laboratory mice (Chen et al., 2015; Sarkar et al., 2019; Guilin et al., 2020; Lee et al., 2020; Mills et al., 2021; Zhuang et al., 2021; Yang et al., 2022; Song et al., 2024). All of these studies used 16S rRNA gene sequences to assess changes in microbiota richness and composition. In one study, MC-LR was administered through intraperitoneal injection of 0.01 mg/kg body mass five times a week for 2 weeks (Sarkar et al., 2019), whereas in the other six studies MC-LR was administered orally through gavage or drinking water. Despite the substantial variation in MC-LR concentration (0.001–0.120 mg/L in drinking water or 0.04–4 mg/kg of body mass every 24–48 h through gavage) and duration of the administration period (1 week to 1 year), all but one study indicated significant changes in abundance of sentinel bacteria (e.g., decline in Firmicutes/Bacteroidetes ratio) in fecal or colonic microbiota. The only study that failed to detect compositional changes in fecal microbiota (Mills et al., 2021), exposed mice to the highest concentration of MC-LR (daily gavage of 4 mg/kg) for the shortest time period (1 week), suggesting that time of exposure may affect detection of microbiota changes. However, an equally short study with lower daily dose (1 mg/kg) reported significant compositional changes in Sprague–Dawley rat (*Rattus norvegicus domestica*) fecal microbiota (Lin et al., 2015). Only two studies sampled cecal microbiota of mice during experimental MC-LR exposure (Chen et al., 2015; Zhuang et al., 2021). In addition to changes in abundance of some bacterial taxa, both studies reported increase in cecal microbiota richness. Increased prokaryotic richness, likely due to colonization by opportunistic pathogens, has been reported for cecal microbiota of prairie grouse (*Tympanuchus spp*.) exposed to crop production (Drovetski et al., 2022) and may represent a common outcome of exposure to environmental stressors.

In the present study, we evaluate effects of an experimental week-long sublethal exposure to purified MC-LR administered through oral gavage on metabolically active prokaryotic microbiota, expression of virulence factors, antimicrobial resistance genes, and metabolic pathways in the ceca of the mallard. To achieve this goal, we employ deep shotgun metatranscriptomics (median of 121,129,328 sequences/sample) of the cecal content of fully-grown juvenile female mallards divided into two exposure categories: birds exposed to a daily dose of 0.75 mg MC-LR (n = 8) and control birds with no MC-LR exposure (n = 8).

## 2 Materials and methods

### 2.1 Experimental design and sampling

We used 16 fully grown juvenile (16–20 weeks old) female mallards raised together on an outdoor farm in Wisconsin, United States (Table 1). During the experiment, all birds were kept in individual cages in the same environmental conditions and fed the same diet. MC-LR doses were prepared by diluting 1 mg/mL MC in >99.95% ethanol, dispensing 0.75 mL of the mixture into a gelatin caplet, and allowing the ethanol to evaporate. Mallards were randomly assigned to two groups: those that received a caplet daily with 0.75 mg MC-LR for 7 days orally (hereafter exposed birds; n = 8) and birds that received a similarly prepared caplet with no MC-LR (hereafter control birds; n = 8). The dosage was confirmed and liver MC-LR concentration was measured in exposed birds (Jenkins et al., 2025). All birds were euthanized approximately 24 h after their last dose.

**TABLE 1:** Mallard (*Anas platyrhynchos*) cecal content sample IDs, sequencing depth, National Center for Biotechnology Information (NCBI) accession numbers, exposure categories (Supplementary Table 1 in Drovetski et al., 2025).

ID	NWHC case number-ID	Number of pair-ended reads	NCBI accession	Exposure category
PI202111	46882-01	69269328	SRX22659392	Control
PI202112	46882-02	59914980	SRX22659393	Control
PI202113	46882-03	74399926	SRX22659400	MC-LR
PI202114	46882-04	63006094	SRX22659401	MC-LR
PI202115	46882-05	61214348	SRX22659402	Control
PI202116	46882-06	65352132	SRX22659403	Control
PI202117	46882-07	73182852	SRX22659404	MC-LR
PI202118	46882-08	57385111	SRX22659405	MC-LR
PI202119	46882-09	64965961	SRX22659406	Control
PI202120	46882-10	49218452	SRX22659407	Control
PI202121	46882-11	54965880	SRX22659394	MC-LR
PI202122	46882-12	49817301	SRX22659395	MC-LR
PI202123	46882-13	53046416	SRX22659396	Control
PI202124	46882-14	59180630	SRX22659397	Control
PI202125	46882-15	58290957	SRX22659398	MC-LR
PI202126	46882-16	66641048	SRX22659399	MC-LR

Immediately after each bird was euthanized, its abdominal cavity was opened with a sterile scalpel blade to expose ceca. One cecum was sliced across with a sterile scalpel blade and approximately 100 mg of cecal content was expelled directly into a 2 mL screw cap tube pre-filled with 1 mL of DNA/RNA Shield and a mix of 0.5 mm and 0.1 mm ultra-high density BashingBeads (Zymo Research, Irvine, CA, USA). Samples were kept at 4 °C until DNA extraction within a maximum of 30 days after collection. Age of birds was confirmed by *bursa Fabricii* presence (well developed, fleshy in juvenile birds, atrophies in adults), and sex was confirmed by gonad examination.

### 2.2 Molecular procedures

Total RNA from the cecal content was extracted using the ZymoBIOMICS DNA/RNA Miniprep Kit (Zymo Research, Irvine, California, USA) following the manufacturer’s instructions. RNA quantity was measured on a Qubit 4.0 using the High-Sensitivity RNA Assay Kit (ThermoFisher Scientific, Waltham, Massachusetts, USA). To test the collection tubes and the extraction kit for possible contamination, we included a single blank sample (a collection tube with 1 mL of DNA/RNA Shield and a mix of 0.5 mm and 0.1 mm ultra-high density BashingBeads but without sample) into our RNA extraction process. The Qubit assay did not detect any RNA using 10 μL of the blank sample (lower detection limit is 0.2 ng/μL), whereas the lowest RNA concentration estimated using 2 μL of our samples was 37.8 ng/μL (median 53.5 ng/μL). We did not sequence this negative control sample as our previous re-sequencing of the negative controls produced inconsistent sets of microbial strains reflecting other samples in the sequencing lane and did not bias microbial composition of real samples containing multiple orders of magnitude greater quantities of nucleic acids.

### 2.3 Shotgun metatranscriptomic sequencing and bioinformatics

RNA extracts were shipped on dry ice to GeneWiz/Azenta Life Sciences (South Plainfield, New Jersey, USA) for quality control and sequencing. RNA libraries were prepared using the NEB Ultra II RNA kit (New England Biolabs, Inc., Ipswich, Massachusetts, USA) and QIAseq FastSelect -rRNA HMR Kit (QIAGEN, Germantown Maryland) for rRNA depletion. To control for possible batch effects, all libraries were sequenced simultaneously on an Illumina HiSeq 4000 platform (2 × 150bp; Illumina, Inc., San Diego, California, USA). The total number of resulting sequences was 1,959,702,832 or 979,851,416 pair-ended reads from our 16 samples. The number of reads obtained from individual samples ranged from 49,218,452 to 74,399,926 with a median of 60,564,664 (Table 1).

Raw sequences were uploaded to the CosmosID Metagenomics Cloud app.cosmosid.com (CosmosID Inc., Germantown, Maryland, USA, www.cosmosid.com) for taxonomic identification, virulence and antimicrobial resistance profiling, and functional analyses. The CosmosID Metagenomics Cloud uses the KEPLER pipeline (CosmosID Hab, 2025c) for host-agnostic microbial taxonomic profiling. KEPLER utilizes k-mer exact-matching and probabilistic alignment to identify and estimate normalized abundance of microbial taxa. Antimicrobial resistance (AMR) genes and virulence factors (VF) are classified using the Resfinder (Florensa et al., 2022) and VFDB (Liu et al., 2022) databases respectively, and the KEPLER-AMR/VF Profiler (CosmosID Hub, 2025a). For metatranscriptomic functional profiling, the quality-controlled and adaptor-trimmed in BBduk (Joint Genome Institute, 2023) reads are translated and searched against a comprehensive and non-redundant protein sequence database, UniRef 90 provided by UniProt (The UniProt Consortium, 2017). The mapping of the reads to genes is weighted by mapping quality, coverage and gene length to estimate community-wide weighted gene family abundances following Franzosa et al. (Franzosa et al., 2018). Gene families are then annotated to MetaCyc (Caspi et al., 2008; Caspi et al., 2018) metabolic pathways (Franzosa et al., 2018) and grouped into gene ontology (GO) terms, carbohydrate active (CAZy) and Enzyme Commission enzymes, and protein domains (Pfam).

We used the normalized read frequency of prokaryotic strains provided by the CosmosID Metagenomics Cloud for statistical analyses. The normalized read frequency is a probabilistic estimate of the number of reads aligning to a reference genome normalized by its size and is suitable for comparative or differential abundance analyses. The normalized read frequencies were cumulative sum scaled (CSS; McMurdie and Holmes, 2014) and log_2_ transformed to account for unequal sequencing depth among samples and non-normal distribution of strains in the samples. Because VFs and AMRs databases are gene-based rather than organism-based, we used the percent total matches (the number of total k-mers identified out of all possible k-mers), which approximates gene abundance in the sample and is directly comparable among samples without normalization. We multiplied these percentages by 10^6^ to produce “copies per million” which we CSS + log_2_ transformed for analyses. For the functional classification, we used copies per million values that represent the Total-Sum Scaled (TSS) abundance values normalized for comparisons across samples with unequal sequencing depth (CosmosID Hub, 2025b). Detailed description of the CosmosID Metagenomics Cloud methodology is provided on the CosmosID Methods page (CosmosID Hab, 2025c).

### 2.4 Data analyses

We compared richness and Shannon Index (Shannon, 1948) values of metabolically active microbial strains, expressed VFs, AMR genes, MetaCyc pathways, Gene Ontology (GO) terms, CAZy and Enzyme Commission enzymes, and Protein families (Pfam) between MC-LR exposure categories (exposed birds and controls; Table 1) using linear model regression (*lm* function) implemented in *Lme4* v. 1.1–36 (Bates et al., 2015) R package and asymptotic test for the equality of coefficients of variation (Feltz and Miller, 1996) implemented in *cvequality* v.0.2.0 (Marwick and Krishnamoorthy, 2019) R package. To compare composition of microbial strains, VFs, AMR genes, MetaCyc pathways, GO Terms, CAZy and Enzyme Commission enzymes, and Pfam between exposure categories, we calculated pairwise Bray-Curtis dissimilarities (Bray and Curtis, 1957) among samples and used them for permutational multivariate analysis of variance PERMANOVA (Anderson, 2001), tests of multivariate dispersion, and principal coordinate analyses (PCoA) implemented in *vegan* v. 2.4–4 (Oksanen et al., 2019) R package. In all analyses listed in this paragraph, we used p ≤ 0.05 as indicative of the null hypothesis rejection.

To identify the microbial strains, VFs, AMR genes, MetaCyc pathways, GO Terms, CAZy and Enzyme Commission enzymes, and Pfam that best explain differences between exposed and control birds, we used the Microbiome Multivariate Associations with Linear Models MaAsLin 3 (Mallick et al., 2021) implemented in R (R Core Team, 2025). Although MaAsLin 3 includes a variety of statistical models for identifying differentially abundant features, we chose the log-transformed linear model on Total Sum Scaled (TSS) strain abundance data but did not normalize other data because they were normalized during processing in the CosmosID Metagenomics Cloud. We used minimum prevalence of 0.25 to exclude features that were found in less than half of the individuals sampled in a single exposure category. We used the maximum false discovery rate corrected *p*-value (*q*-value) of 0.05.

## 3 Results

### 3.1 Prokaryotes

The Kepler pipeline identified a total of 263 metabolically active prokaryotic strains in our dataset - two archaeons and 261 bacteria (Supplementary Table 2 in Drovetski et al., 2025). Archaea were represented by a mesophilic ammonia-oxidizing chemolithoautotroph *Nitrososphaera viennensis* (Thermoproteota, 3.2% of total abundance, detected in all 16 birds) (Stieglmeier et al., 2014) and a methanogen *Methanobrevibacter_A woesei* (Methanobacteriota, 0.7%, detected in two exposed and two control birds) (Miller and Lin, 2002). Bacteria were represented by 11 phyla: Bacteroidota (37.9% of total abundance; detected in all 16 birds), Bacillota_C (29.1%, n = 16), Spirochaetota (10.80%, n = 16), Actinomycetota (5.92%, n = 16), Bacillota_A (5.90%, n = 15), Bacillota (2.37%, n = 14), Desulfobacterota (1.87%, n = 15), Pseudomonadota (1.64%, n = 16), Fusobacteriota (0.49% control n = 4, exposed n = 3), Campylobacterota (0.06%, n = 1 in each exposure category), and Deferribacterota (0.05%, control n = 2, exposed n = 3).

**FIGURE 1:**
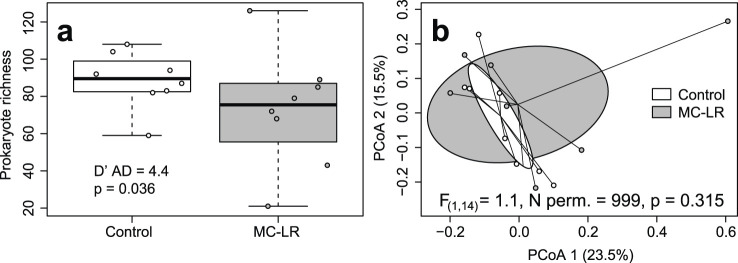
Boxplot depicting richness **(a)** and principal coordinates analysis (PCoA) plot based on Bray-Curtis dissimilarities **(b)** of metabolically active prokaryotic strains in ceca of mallards (*Anas platyrhynchos*) experimentally exposed to microcystin-LR (MC-LR) versus controls. Boxes in the panel **(a)** represent interquartile ranges (from lower quartile to the upper quartile), whiskers represent minimum and maximum values, and the thick horizontal lines represent medians. Ellipses in panel **(b)** represent one standard deviation around the centroid. The equality of richness coefficients of variation **(a)** was rejected, whereas equality of multivariate dispersion **(b)** was not. The value of *D*′*AD* test statistic measures how far each sample coefficient of variation is from estimated population coefficient of variation.

Prokaryotic richness in individual samples varied from 21 to 126 strains with a median of 84. Control birds had higher median richness (89.5) than exposed birds (75.5) but this difference was not statistically supported (Adj. *R* ^2^ = 0.041, F_(1, 14)_ = 1.638, p = 0.222; Figure 1a), apparently due to 2.5 times greater coefficient of richness variation in exposed birds (0.430) than that in controls (0.171; D’AD = 4.405, p = 0.036). Shannon Index values varied from 1.611 to 3.565, with a median of 2.829. Neither Shannon Index values nor their coefficients of variation differed between exposed and control birds (Adj. *R* ^2^ = −0.052, F_(1, 14)_ = 0.256, p = 0.256; D’AD = 0.565, p = 0.452).

Exposure to MC-LR did not affect the composition of the metabolically active prokaryotic community (PERMANOVA *R* ^2^ = 0.042, F_(1, 14)_ = 0.617, p = 0.954), and the two exposure groups overlapped substantially on the PCoA plot (Figure 1b). Although the mean distance to centroid was higher in the MC-LR exposed birds (0.392) than in controls (0.336), the null hypothesis of the homogeneity of multivariate dispersions between exposure groups was not rejected (F_(1, 14)_ = 1.081, p = 0.315). MaAsLin3 did not identify any prokaryotic strains differentially active between control and exposed birds.

### 3.2 Virulence factors

KEPLER-AMR/VF Profiler identified a total of 102 expressed VFs in our dataset (Supplementary Table 3 in Drovetski et al., 2025). Richness of expressed VFs varied from 1 to 48 with a median of 7.5. No relationship between exposure to MC-LR and VF richness (Adj. *R* ^2^ = −0.049, F_(1, 14)_ = 0.293, p = 0.597) or difference in coefficient of richness variation (D’AD = 0.003, p = 0.953) between exposed and control birds was observed. Shannon Index values varied from 0 to 3.779, with a median of 1.908. Shannon Index values did not differ between exposed and control birds (Adj. *R* ^2^ = −0.071, F_(1, 14)_ = 0.006, p = 0.942) and the null hypothesis of equality of their coefficients of variation was not rejected (D’AD = 1.044, p = 0.307). VFs load (the sum of all VFs total matches in the sample) varied from 0.105 to 23.907, with a median of 1.585. Neither the VF load (Adj. *R* ^2^ = −0.055, F_(1, 14)_ = 0.216, p = 0.649) nor its coefficients of variation (D’AD = 0.023, p = 0.881) differed between exposed and control birds. Exposure to MC-LR did not affect the composition of expressed VFs (PERMANOVA *R* ^2^ = 0.053, F_(1, 14)_ = 0.776, p = 0.808), and the two exposure groups overlapped substantially on the PCoA plot. Although the mean distance to centroid was higher in the MC-LR exposed birds (0.622) than in controls (0.592), the null hypothesis of the homogeneity of multivariate dispersions between exposure groups was not rejected (F_(1, 14)_ = 0.443, p = 0.477). MaAsLin3 identified no VFs whose expression differed between exposed and control birds.

### 3.3 Antimicrobial resistance genes

**FIGURE 2:**
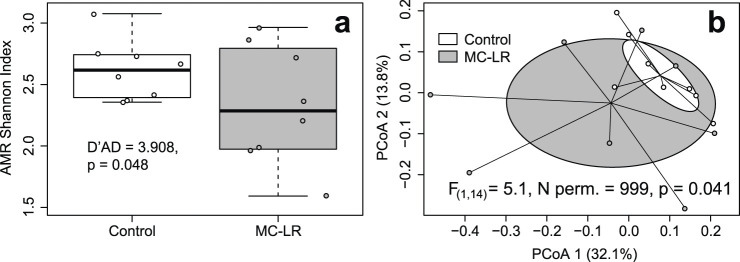
Boxplot depicting the Shannon Index **(a)** and principal coordinates analysis (PCoA) plot based on Bray-Curtis dissimilarities **(b)** of expressed antimicrobial resistance genes (AMRs) in ceca of mallards (*Anas platyrhynchos*) experimentally exposed to microcystin-LR (MC-LR) versus controls. Boxes in the panel **(a)** represent interquartile ranges (from lower quartile to the upper quartile), whiskers represent minimum and maximum values, and the thick horizontal lines represent medians. Ellipses in panel **(b)** represent one standard deviation around the centroid. Both the equality of richness coefficients of variation **(a)** and equality of multivariate dispersion **(b)** were rejected. The value of *D*′*AD* test statistic measures how far each sample coefficient of variation is from estimated population coefficient of variation.

KEPLER-AMR/VF Profiler identified a total of 67 expressed AMRs from nine classes in our dataset (Supplementary Table 4 in Drovetski et al., 2025). Richness of expressed AMRs varied from 6 to 25 with a median of 15.0. There was no relationship between exposure to MC-LR and richness of AMRs (Adj. *R* ^2^ = −0.022, F_(1, 14)_ = 1.334, p = 0.267). There was no difference in coefficient of richness variation between exposed birds and controls (D’AD = 1.956, p = 0.162). Shannon Index values varied from 1.592 to 3.077, with a median of 2.491. Shannon Index values did not differ between exposed and control birds (Adj. *R* ^2^ = 0.073, F_(1, 14)_ = 2.186, p = 0.161), whereas the null hypothesis of equality of their coefficients of variation was rejected (control cv = 0.093, exposed cv = 0.209; D’AD = 3.908, p = 0.048; Figure 2a). The load of expressed AMRs varied from 2.634 to 14.009, with a median of 6.452. Neither the load (Adj. *R* ^2^ = −0.025, F_(1, 14)_ = 0. 0.641, p = 0.437) nor its coefficients of variation (D’AD = 0.813, p = 0.367) differed between exposed and control birds. Exposure to MC-LR did not affect the composition of expressed AMRs (PERMANOVA *R* ^2^ = 0.082, F_(1, 14)_ = 1.246, p = 0.216), and the two exposure groups overlapped completely on the PCoA plot (Figure 2b). The mean distance to centroid was higher in the MC-LR exposed birds (0.362) than in controls (0.266; F_(1, 14)_ = 5.053, p = 0.035) rejecting the null hypothesis of the homogeneity of multivariate dispersions between exposure groups. MaAsLin3 identified no AMRs differentially expressed between exposed and control birds.

### 3.4 Metabolic pathways

**FIGURE 3:**
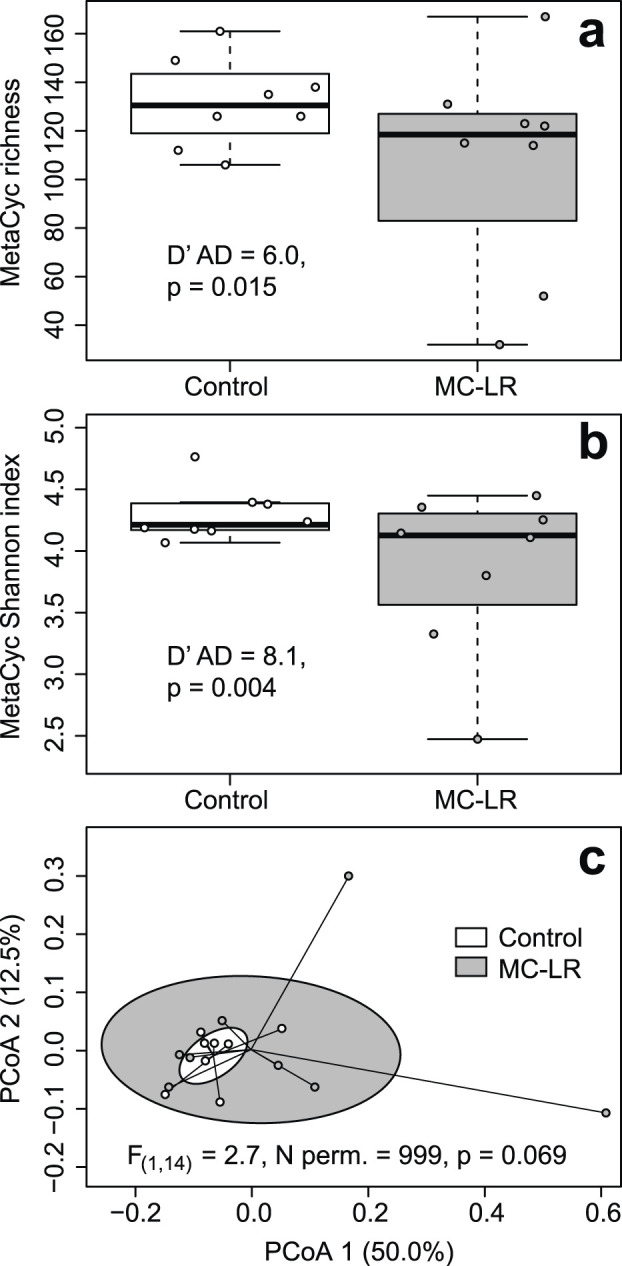
Boxplots depicting richness **(a)** Shannon Index **(b)** and principal coordinates analysis (PCoA) plot based on Bray-Curtis dissimilarities of expressed metabolic (MetaCyc) pathways **(c)** in ceca of mallards (*Anas platyrhynchos*) experimentally exposed to microcystin-LR (MC-LR) versus controls. Boxes in the panel **(a, b)** represent interquartile ranges (from lower quartile to the upper quartile), whiskers represent minimum and maximum values, and the thick horizontal lines represent medians. Ellipses in panel **(c)** represent one standard deviation around the centroid. The equality of coefficients of variation of richness and Shannon Index **(a,b)** and equality of multivariate dispersion **(c)** were rejected, although marginally for the latter. The value of *D*′*AD* test statistic measures how far each sample coefficient of variation is from estimated population coefficient of variation.

CosmosID Metagenomics Cloud identified a total of 242 expressed MetaCyc pathways in our dataset (Supplementary Table 5 in Drovetski et al., 2025). Their richness varied from 32 to 167 with a median of 124.5. The richness of expressed MetaCyc pathways was greater in controls (median = 130.5) than exposed (118.5) birds but this differences was not statistically supported (Adj. *R* ^2^ = 0.072, F_(1, 14)_ = 2.157, p = 0.164), likely due to three fold greater coefficient of richness variation in exposed (cv = 0.409) than in control birds (cv = 0.138; D’AD = 5.957, p = 0.015; Figure 3a). Shannon index values varied from 2.474 to 4.764. Similar to differences in richness, Shannon Index values were greater in controls (median = 4.213) than in exposed birds (median = 4.127) but this difference was marginally supported (Adj. *R* ^2^ = 0.120, F_(1, 14)_ = 3.049, p = 0.103) also likely due to over three fold greater coefficients of variation in exposed birds (cv = 0.172) than in controls (cv = 0.051; D’AD = 8.073, p = 0.004; Figure 3b). Exposure to MC-LR did not alter composition of expressed MetaCyc pathways (PERMANOVA *R* ^2^ = 0.087, F_(1, 14)_ = 1.339, p = 0.203), and the exposure groups overlapped completely on the PCoA plot (Figure 3c). The mean distance to centroid was greater in the MC-LR exposed birds (0.265) than in controls (0.171), but the null hypothesis of the homogeneity of multivariate dispersions between exposure groups was marginally rejected (F_(1, 14)_ = 2.707, p = 0.059). MaAsLin3 identified no MetaCyc pathways that were differentially expressed between exposed and control birds.

### 3.5 Gene ontology terms (GO terms), protein families (Pfam), Enzyme Commission enzymes, and carbohydrate active enzymes (CAZy)

**FIGURE 4:**
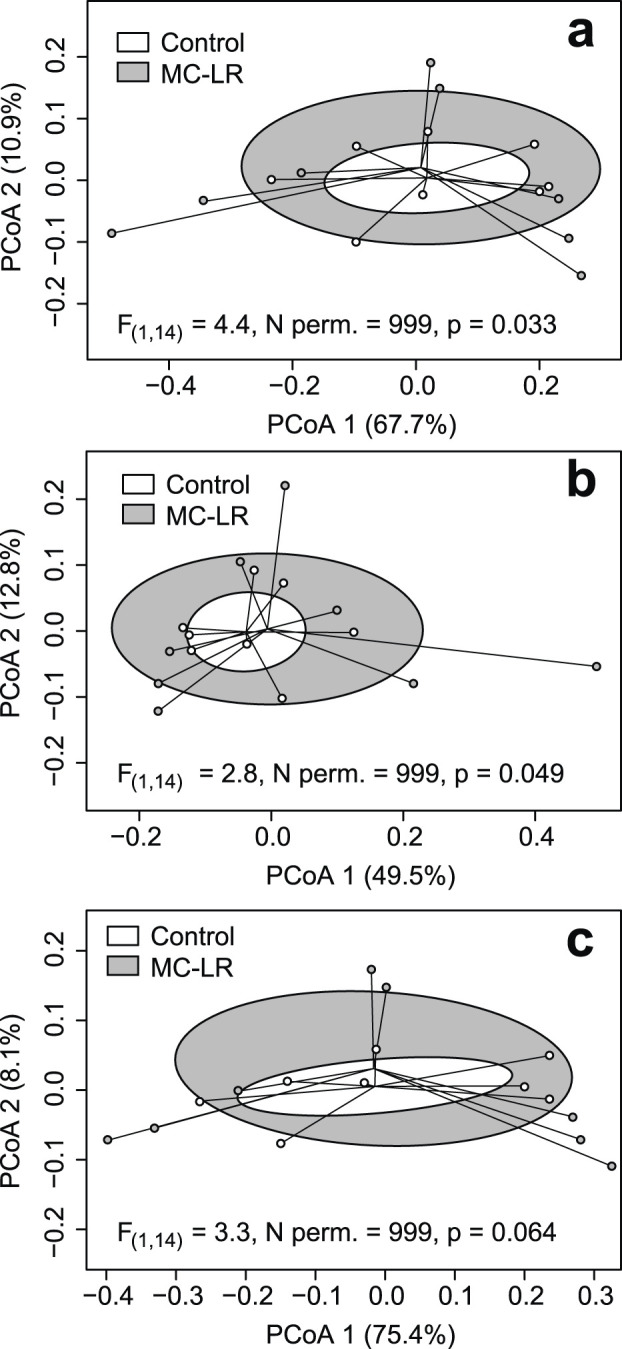
Principal coordinates analysis (PCoA) plots based on Bray-Curtis dissimilarities of expressed protein families Pfam; **(a)** Enzyme Commission enzymes **(b)** and gene ontology (GO) terms **(c)**. Ellipses represent one standard deviation around centroid. In all cases the equality of multivariate dispersion was rejected, although marginally for the latter.

There were no differences in α-diversity measurements, their coefficients of variation, or in composition of GO terms, CAZy and Enzyme Commission enzymes, and Pfam (Supplementary Tables 6–9, respectively in Drovetski et al., 2025) between control and MC-LR exposed birds. MaAsLin 3 did not identify any features that were differentially expressed between the experimental groups. However, β-diversity multivariate dispersion was greater in the MC-LR exposed birds than in controls for Pfam (F_(1,14)_ = 4.4, N permutations = 999, p = 0.033; Figure 4a) and Enzyme Commission enzymes (F_(1, 14)_ = 2.8, N permutations = 999, 0.049; Figure 4b), and the null hypothesis of the equality of multivariate dispersion was marginally rejected for GO terms (F_(1, 14)_ = 3.3, N permutations = 999, p = 0.064; Figure 4c). Only for carbohydrate active enzymes, the equality of multivariate dispersion was not rejected (F_(1, 14)_ = 2.3, n permutations = 999, p = 0.132).

## 4 Discussion

The goal of our study was to investigate changes in mallard cecal microbiota activity and function following a week-long, orally administered exposure to a relatively low, sublethal dose of purified MC-LR. We chose mallard for this experimental study because this species is widely used in toxicity experiments and is one of the most frequently affected during wildlife mortality events coinciding with cyanobacterial blooms (Rattner, 2022). Mallard’s abundance, ecology, and foraging habits routinely put them in contact with algal blooms and facilitate ingestion of cyanotoxins. To the authors’ knowledge, this is the first study of MC-LR exposure effects on avian microbiome and the first to use shotgun RNA sequencing in terrestrial wildlife.

Neither richness, Shannon index values, nor composition of metabolically active prokaryotes, expressed VFs, AMRs, MetaCyc pathways, GO terms, Pfam, Enzyme Commission nor CAZy enzymes differed between exposed and control birds suggesting a lack of deterministic shifts in composition and function of mallard cecal microbiota in response to short-term, low-dose MC-LR exposure. Likewise, there were no metabolically active strains, VFs, AMRs, metabolic pathways, GO terms, enzymes or proteins that were differentially active between exposed and control birds. This appears to contradict findings of both earlier murine studies of MC-LR effects on cecal prokaryotic microbiota based on denaturing gradient gel electrophoresis (Chen et al., 2015) or short amplicon sequencing (Zhuang et al., 2021) of 16S rRNA gene. Both studies reported increases in prokaryotic microbiota richness, Shannon index values, and changes in abundance of sentential cecal bacteria. However, during library preparation in our study, the 16S rRNA gene fragments were depleted. It is plausible that an increase in operational taxonomic units (OTU) richness identified using substitutions and indels in hypervariable segments of 16S rRNA gene does not necessarily translate into proportional increases in strain richness calculated using variation in expressed non-ribosomal mRNA.

The lack of deterministic response, however, does not mean there was no effect of MC-LR exposure on the mallard cecal microbiota and its function. We observed increase in variance of α-diversity indicators and/or β-diversity multivariate dispersion of metabolically active prokaryotes and their expressed AMRs, MetaCyc pathways, GO terms, enzymes and proteins in exposed birds. Only VFs and carbohydrate active enzymes did not display elevated stochasticity in the birds exposed to MC-LR. The elevated stochasticity in the cecal microbiota and its function of exposed birds relative to controls, observed in our data, is consistent with the Anna Karenina Principle (Zaneveld et al., 2017) that has been documented in a variety of animal and plant study systems (Zaneveld et al., 2017; Ahmed et al., 2019; Ma, 2020; Arnault et al., 2023; Li and Yang, 2025). This principle, when applied to host-associated microbiota, suggests that in response to stressors, symbiotic microbial community composition may not shift to a new stable state but rather become unstable leading to its greater variation among stressor-exposed than unexposed hosts. Stressor-related loss of the ability to regulate microbial community composition by the host and/or its microbiota and resulting dysbiosis, the disruption of microbiota homeostasis, have been linked to many host diseases, adverse outcomes, and reduced fitness across multicellular eukaryotes from plants and corals to humans (Zaneveld et al., 2017; Ahmed et al., 2019; Ma, 2020; Arnault et al., 2023; Li and Yang, 2025). Unfortunately, we cannot determine whether the observed disruption of microbiome homeostasis is a direct effect of microbiota contact with the toxin or might be combined with indirect effect(s) through modulation of some host organ(s)’ function because our understanding of MC-LR toxicity in birds is still in its infancy (Rattner, 2022). Even in much better studied mammals, we have little information on MC-LR toxicokinetics and toxicodynamics besides liver and male reproductive system (Ma et al., 2021).

Disruption of cecal microbiota homeostasis is likely to be particularly consequential to the host. In predominantly herbivorous waterfowl, including mallard and gamebirds, paired ceca function as microbial fermentation chambers that break up indigestible complex carbohydrates (i.e., dietary fiber) into short chain fatty acids that serve as the primary source of energy to the host and convert uric acid to ammonia (Clench and Mathias, 1995; Svihus et al., 2013; Svihus, 2014). Strong functional selection and long digesta retention time in the cecum greatly reduce interindividual α- and β-diversity variance in cecal microbiota relative to microbiotas of all other gut regions (Drovetski et al., 2018; Drovetski et al., 2019). Anna Karenina effects, i.e., stochastic responses to sublethal MC-LR exposure, which we observed in the mallard cecal microbiota, have the lowest tolerance limits but greatest effects on host energy balance and, ultimately, fitness than similar responses in other digestive tract regions.

In conclusion, our study revealed that short-term sublethal oral exposure to purified MC-LR affects cecal microbiome in the semi-domestic mallard. These effects are consistent with the Anna Karenina Principle postulating that microbiota of the host exposed to a stressor, may have a stochastic rather than deterministic response. Indeed, the coefficient of variation of the metabolically active microbiota richness was greater in the birds exposed to MC-LR than in controls. At the same time there were neither shifts in cecal microbiota richness, Shannon Index, composition nor differentially active strains between exposure groups. However, manifestation of the Anna Karenina Principle was even more pronounced in microbiota function. Increased stochasticity among exposed birds was detected in richness, Shannon Index, and composition of expressed metabolic pathways, resulting in increased multidimensional dispersion of expressed AMRs, GO terms, proteins and enzymes. To the authors’ knowledge, this is the first documented extension of the Anna Karenina Principle beyond community composition to its function, which has been implied previously (Zaneveld et al.,2017; Arnault et al., 2023) but not tested.

## Data Availability

Publicly available datasets were analyzed in this study. These data can be found here: https://doi.org/10.5066/P1OCUFM6. Raw sequence archives were deposited to the National Center for Biotechnology Information as the bioproject PRJNA1045981: https://www.ncbi.nlm.nih.gov/bioproject/PRJNA1045981/.
